# Factors Regulating Immunoglobulin Production by Normal and Disease-Associated Plasma Cells

**DOI:** 10.3390/biom5010020

**Published:** 2015-01-21

**Authors:** David A. Jackson, Sherine F. Elsawa

**Affiliations:** Department of Biological Sciences, Northern Illinois University, 349 Montgomery Hall, DeKalb, IL 60115, USA; E-Mail: djackson10@niu.edu

**Keywords:** immunoglobulin, B cells, plasma cells, malignant B cells, autoimmune B cells

## Abstract

Immunoglobulins are molecules produced by activated B cells and plasma cells in response to exposure to antigens. Upon antigen exposure, these molecules are secreted allowing the immune system to recognize and effectively respond to a myriad of pathogens. Immunoglobulin or antibody secreting cells are the mature form of B lymphocytes, which during their development undergo gene rearrangements and selection in the bone marrow ultimately leading to the generation of B cells, each expressing a single antigen-specific receptor/immunoglobulin molecule. Each individual immunoglobulin molecule has an affinity for a unique motif, or epitope, found on a given antigen. When presented with an antigen, activated B cells differentiate into either plasma cells (which secrete large amounts of antibody that is specific for the inducing antigen), or memory B cells (which are long-lived and elicit a stronger and faster response if the host is re-exposed to the same antigen). The secreted form of immunoglobulin, when bound to an antigen, serves as an effector molecule that directs other cells of the immune system to facilitate the neutralization of soluble antigen or the eradication of the antigen-expressing pathogen. This review will focus on the regulation of secreted immunoglobulin by long-lived normal or disease-associated plasma cells. Specifically, the focus will be on signaling and transcriptional events that regulate the development and homeostasis of long-lived immunoglobulin secreting plasma cells.

## 1. Introduction

Immunoglobulins (Igs) are key molecules for an effective immune response to human pathogens. Characteristics of these proteins allow the immune system to recognize and respond to a myriad of pathogens effectively [1]. Ig or antibody (Ab) secreting cells develop from B cells, which undergo selection processes during development in the bone marrow that lead to expression of a single antigen (Ag)-specific Ig. The Ig produced by these individual B cells has an affinity for a unique motif found on a given Ag [2]. When presented with Ag, activated B cells, in turn, differentiate into either plasma cells (PCs), which secrete large amounts of Ab specific for the pathogenic Ag, or memory B cells that are long-lived and illicit a stronger and faster response if the host is reinfected with the same pathogen in the future [3]. Secreted Ig bound to Ag serves as an effector molecule that directs other cells of the immune system in the eradication of the specific pathogen in the host organism [1]. This review will focus on the regulation of secreted Ig by long-lived PCs both in the context of normal physiology and disease states. Specifically, the focus will be on signaling and transcriptional events that regulate the development and homeostasis of long-lived Ig secreting PCs.

Naïve B cells, or B cells that have undergone development and are now circulating in secondary lymphatic tissues, express both membrane-bound IgM (mIgM) and IgD (mIgD). Both mIgM and mIgD together with Igα and Igβ function as B cell receptors for naïve B cells [4]. Naïve B cells have already undergone V(D)J recombination during development and both mIgM and mIgD on a given B cell is specific for the same Ag [5,6]. Likewise, upon encounter with Ag and subsequent activation, which is discussed in greater detail below, B cells either differentiate into short-lived PCs, or enter the germinal center (GC) where they undergo affinity maturation and class switch recombination (CSR) and differentiate into either long-lived PCs or memory B cells [7,8].

Regulation of the production of Ig is highly important for an effective immune response and dysregulation of Ig production is characteristic of several diseases, such as Sjӧgren’s syndrome, multiple myeloma, and Waldenstrӧm macroglobulinemia [9,10,11]. A thorough understanding of the processes involved in both proper regulation and dysregulation of Ig production is paramount to the future development of novel therapies for these diseases.

## 2. B Cell Development and Activation

During B cell development, genes for the light chain and heavy chain of Ig undergo recombination which confers the specificity for a given Ag [6]. During this process, B cells are subjected to multiple rounds of selection, both in the bone marrow (BM) and in secondary lymphoid tissues, which ensures that mature B cells will possess Ig that is reactive with a single Ag and that is not autoreactive [12,13].

Upon encountering Ag, B cells become activated. Depending on the nature of the Ag, B cell activation is carried out with the help of CD4^+^ (T_H_) cells (TD; T-dependent activation) or without the help of T_H_ cells (TI; T-independent activation) [14]. Nearly all, high affinity Ab producing, long-lived PCs are generated by mechanisms involving T_H_ cells and are therefore the result of TD responses [15]. During a TD response, activated B and T cells remain complexed for 3 days and an exchange of signals, such as CD40/CD40L, leads to initiation of one of two fates for the B cell. Some naïve B cells move into the extrafollicular region of the lymph node, where they differentiate into short-lived PCs that produce only IgM as an initial response to infection. Others migrate into the follicles where they form GCs [16]. GCs are scaffolded with follicular dendritic cells (FDCs). These follicular dendritic cells function as Ag presenting cells (APCs) and provide cytokine and chemokine signals along with a special subset of T-cells, T follicular helper cells (T_FH_) which facilitate GC reactions [17,18]. The GC is the site where B cells undergo affinity maturation, clonal expansion, and ultimately differentiate into either high affinity, long lived PCs or memory B cells. GC reactions are carried out through a complex orchestration of fluctuating transcription factors driven by B cell interactions with FDCs, T_FH_ and the cytokines they produce.

## 3. Role of the Germinal Center in PC Differentiation

GCs are important in the regulation of Ig, as they are the site of somatic hypermutation (SHM), which leads to the formation of high affinity Ab, and one of the sites of class switch recombination (CSR), which leads to a change in effector functions of secreted Abs [1,7]. Although it will not be covered further in this review, it should be noted that CSR has also been shown to occur during extrafollicular B cell differentiation due to exposure to TI antigen [19,20]. Through the research conducted over the past two decades, a handful of what have been termed master regulators of GC reactions and subsequent PC differentiation have been identified. These transcription factors include B cell lymphoma 6 (BCL6), interferon regulatory factor 4 (IRF4), B lymphocyte-induced maturation protein-1 (BLIMP-1) (induced by *Prdm1* gene and referred to as BLIMP-1 from here on), and X-box binding protein-1 (XBP-1) [17,21,22,23,24,25,26]. Regulation of these transcription factors is facilitated by interactions with cytokines and cell-cell signaling within the GC. In turn, these master regulators activate numerous downstream target genes that result in affinity maturation, clonal expansion, and eventual exit from the GC and differentiation into long-lived, high affinity Ab secreting cells.

## 4. Transcriptional Regulation of GC Reactions and PC Differentiation

BLIMP-1 is required for differentiation of B cells into PCs and the subsequent secretion of Ig. It is often referred to as the “master regulator” of PC differentiation. Mice with BLIMP-1 knockout that have been exposed to Ag designed to induce a TD immune response, have extremely low levels of PCs generated and also have significantly decreased expression of secreted heavy chain mRNA [27]. BLIMP-1 is also expressed at high levels in long-lived PCs residing in the BM and, in fact, is required to maintain long-lived PC populations [28]. Discussed below, are a number of factors both upstream and downstream of BLIMP-1 activation that lead to the PC phenotype and high volume Ig secretion.

In the early stages of GC development, BCL6 is upregulated and required for the development of GCs [29]. Repression of BLIMP-1 by BCL6 prevents premature differentiation of GC B cells [30]. Another factor that appears to be involved in BCL6 mediated repression of BLIMP-1 is the basic leucine zipper transcription factor 2/BTB and CNC homology-1 (BACH2). BACH2 and BCL6 have been shown to negatively regulate BLIMP-1 separately in experiments using homozygous knockout mice models [29,31]. Mice with these homozygous knockouts have severely impaired ability to form GCs in response to TD Ag and there was disproportionate increase in short-lived, IgM secreting PCs in these mice compared with wild-type (WT) controls [31]. Recently, mice with heterozygous knockout for BACH2 or BCL6 demonstrated only a mild decrease in GC formation and no difference in PC populations after TD Ag encounter compared to WT mice [32]. Interestingly, mice with heterozygous knockout of both BCL6 and BACH2 had much higher IgM secreting PC populations than in WT controls after being treated with TD Ag. Furthermore, mRNA analysis revealed increased expression of both BLIMP-1 and XBP-1 suggesting a cooperative role between BCL6 and BACH2 in preventing premature PC differentiation during GC development [32].

A handful of signaling pathways and transcription factors have been shown to upregulate BLIMP-1 during the GC reaction and subsequently lead to PC differentiation. The B cell specific coactivator OBF-1 (also called BOB1 and OCA-1) was shown to be a positive regulator of BLIMP-1 using an OBF-1 knockout model in mice [33]. OBF-1 knockout mice had a significant decrease in IgG secretion and a modest decrease in IgM secretion. Analysis of mRNA revealed a decrease in BLIMP-1 transcripts in OBF-1 knockout mice compared to control mice. Of note is the fact that OBF-1 knockout failed to have an effect on PC differentiation in TI Ag stimulated PC differentiation suggesting that OBF-1 is differentially regulated in the TI response [33]. Another pathway recently shown to be involved in BLIMP-1 activation is the extracellular regulated MAP kinase/mitogen-activated protein kinase (ERK/MAPK) pathway. Mice with a conditional ERK2 knockout had decreased levels of BLIMP-1 [34]. Furthermore, it appears that the mechanism for this regulation of BLIMP-1 through ERK may involve downregulation of the BLIMP-1 repressor, BACH2 [35]. The nuclear factor kappa B (NF-κB) pathway also plays a role in BLIMP-1 regulation. Mice with conditional knockout of v-Rel avian reticuloendotheliosis viral oncogene homolog A (RelA) demonstrated decreased BLIMP-1 transcript and protein levels [36]. Another positive regulator of BLIMP-1 is, signal transducer and activator of transcription 3 (STAT3), one of the downstream effectors of cytokine signaling [21]. STAT3 overexpression in human B cells stimulated with CD40 ligand (CD40L) and Ag resulted in an increase in BLIMP-1, IRF4, and XBP-1 expression [21]. Interestingly, co-transducing cells with both STAT3 and BCL6 resulted in a PC intermediate with a proliferative phenotype and high volume Ig secretion [21].

Multiple studies have shown IRF4 to be necessary for CSR and PC differentiation [37,38,39]. IRF4 appears to positively regulate BLIMP-1 and in its absence, the entire BLIMP-1 mediated PC differentiation program does not proceed [39]. A recent study has shown that IRF4 and STAT3 cooperatively activate BLIMP-1 in the late GC/early plasmablast stages of PC differentiation [24]. However, the exact role of IRF4 in GC formation is unclear. Conflicting studies have emerged in the last few years proposing that IRF4 is dispensable in B cells for GC development and others maintaining that it is indispensable in B cells for GC formation [17,25]. IRF4 appears to be clearly required in the GC reaction and subsequent differentiation into PCs, but the exact role IRF4 in GC formation, whether through a B cell intrinsic mechanism or T cell intrinsic mechanism, will require further investigation.

A bidirectional relationship exists between BLIMP-1 and BCL6. Although BCL6 is one of the downstream targets of BLIMP-1 repression, it is also one of its upstream regulators. The transcriptional relationship between BLIMP-1 and BCL6 is one of mutual repression and multiple studies have shown a modulation of levels of BCL6 and BLIMP-1 throughout the GC reaction [21,28]. Initially in the GC reaction, BCL6 levels are high enough to effectively repress BLIMP-1. As the reaction proceeds, factors such as IRF4, which activates BLIMP-1, leads to gradually increasing levels of BLIMP-1. Eventually, a tipping point is reached where BLIMP-1 can effectively silence BCL6 and the GC B cell enters the PC differentiation program [21]. Furthermore, the study showed a role for STAT3 as a direct activator of BLIMP-1, as well as being a competitive inhibitor of BCL6-mediated repression of BLIMP-1. This is accomplished by STAT3 binding to a 3' intron of BLIMP-1 at the site of BCL6 binding [21]. Ultimately this leads to transcriptional activation of BLIMP-1 by STAT3.

Another downstream target of BLIMP-1 is PAX-5. PAX-5 is a key transcription factor in B cell development and fate determination of lymphoid progenitors in the BM. It is a definitive B cell marker and maintains the B cell phenotype [40]. The repression of PAX-5 by BLIMP-1 de-represses factors necessary for PC differentiation, such as XBP-1, J-chain, IGH, and IGL [41]. 

XBP-1 is a component of one of the arms of the unfolded protein response (UPR). The UPR functions to relieve endoplasmic reticulum (ER) stress upon accumulation of unfolded proteins [42]. There are three regulatory arms of the UPR. These are carried out by the proteins, protein kinase RNA activated (PKR)-like ER kinase (PERK), activating transcription factor 6 (ATF6a), and inositol-requiring enzyme-1 (IRE1) [43], which ultimately activate a myriad of factors from chaperone proteins to protein trafficking proteins to calcium modulators and if necessary, apoptotic proteins [43]. XBP-1 is a component of the IRE1 branch of the UPR. IRE1 splices the transcript of XBP-1, yielding it active (XBP-1s) [42].

It should not be surprising that the UPR is involved in PC development given the switch to a high efficiency Ig secreting cell and the notable morphological changes that accompany differentiation, namely the greatly expanded ER. Studies have shown that the PERK branch and the ATF6a branch of the UPR are not involved in Ig secretion [44,45]. Likewise, XBP-1 has been implicated as a required factor in the generation of Ab secreting cells over the last two decades. Mice lacking XBP-1 had lower serum Ig levels than WT mice [26]. It was further shown using murine B cell lymphoma cells, that activation using cytokines and lipopolysaccharide (LPS) resulted in increased XBP-1 transcript levels which correlated with increased IgM [46]. Further, XBP-1 was not required for initial mu (μ) chain synthesis, but 2 days after activation, XBP-1 deficiency resulted in a sharp decrease in Ig production [47]. In contrast, XBP-1 had no effect on light chain synthesis [47]. XBP-1’s requirement for Ig secretion was followed by the discovery of a connection between BLIMP-1 and XBP-1. A study using murine splenic cells revealed XBP-1 to be downstream of BLIMP-1 in the PC differentiation program [48]. The study also showed that XBP-1 regulated genes involved in ER expansion [48]. There is some question as to whether XBP-1 has an effect on other aspects of PC differentiation or only regulates Ig secretion. One study showed a modest decrease in CD138^+^ (PC surface marker) cells using XBP-1 conditional knockout mice [49]. Not only were PC numbers diminished but PCs failed to colonize to the bone marrow. On the other hand, another study published the same year showed, *in vivo*, that differentiation of activated B cells to CD138^+^ cells still occurred but these cells failed to fully differentiate to Ig secreting PCs [50]. The study also showed no effect of XBP-1 on memory B-cell formation [50]. Similarly, another study using conditional knockout mice showed that XBP-1 deletion had no effect on differentiation of activated B cells into CD138^+^ PCs but did result in decreased Ig producing cells when compared with WT mice [51]. Furthermore, using a BLIMP-1 GFP mutant reporter mouse, the study showed that the effects of XBP-1 on Ig secretion were most notable in bone marrow (BM) PCs expressing high levels of BLIMP-1 [51]. All three of the above studies, used mice with cre-recombinase under the control of the CD19 promoter, in order to generate B cell specific knockouts [49,50,51]. Finally, very recently, the interplay between XBP-1 and its activator IRE1 through a process known as regulated IRE1 dependent decay (RIDD) was examined [52]. During a UPR, if an effective response is not mounted to relieve ER stress, IRE1 will begin splicing transcripts of the respective unfolded protein accumulating in the ER [53]. In this study, a negative feedback loop where the spliced form of XBP-1 represses IRE1 activity was demonstrated. More importantly, the study also showed that in the absence of XBP-1, IRE1 will splice μS transcript resulting in decreased IgM secretion and an impaired immune response [52]. To date, the requirement of XBP-1 has been clearly demonstrated and appears to be solely responsible for factors involved in the secretory machinery of these professional Ab secreting cells.

## 5. Cytokines in the GC Reaction and PC Differentiation

As discussed above, there are a number of transcriptional and physiological events that are finely regulated in order for PC generation from GC B cells to occur. Orchestrating these events is the cytokine crosstalk between the cells composing the GC, namely centrocytes, centroblasts, follicular helper T cells (T_FH_) and follicular dendritic cells. The exact timing and concentrations of paracrine and autocrine signals necessary for appropriate development of the GC, proliferation, affinity maturation, class-switch recombination, and eventually differentiation into long lived PC or memory B cells is yet to be fully unraveled. However, there have been numerous studies over the last 30 years, many *in vitro* with some *in vivo*, that demonstrate the abilities of different cytokines to enhance GC development and push differentiation towards the PC phenotype and subsequent increased Ig secretion.

Interleukin-2 (IL-2) has long been associated with, and believed to be required for, effective GC formation and subsequent PC differentiation [54]. Early mouse studies established IL-2 as a factor present in the GC reaction, as it was found to be abundantly secreted by activated T cells [54]. Furthermore, these early studies determined that blockade of IL-2 using IL-2 specific Ab (αIL-2 Ab) resulted in decreased Ig secretion when B cells were cocultured with activated T-cells [54]. More recently, IL-2 was shown to have an effect in human lymphocytes in patients exposed to hepatitis B virus (HBV) vaccine. Similar to mice data, activated human T cells produced elevated levels of IL-2 upon encounter with Ag and blockade of IL-2 with αIL-2 Ab resulted in not only decreased levels of Ig but of Ig producing cells [55]. Some evidence points to IL-2 as not being as essential as earlier thought, when compared to another cytokine, IL-21 [56]. However, a recent study demonstrated that early priming (within 2 days) of activated murine B cells with IL-2 resulted in maximum PC differentiation via downregulation of BACH2 through an ERK dependent mechanism [35]. In that study, substituting IL-21 for IL-2 did not yield the same results nor was ERK activation seen in cells treated with IL-21 [35]. Therefore, IL-2 appears to be at least an enhancer of PC generation.

Another cytokine involved in the GC reaction and activated B cell differentiation is IL-4. *In vitro* studies using human B cells show that IL-4, when used in combination with requisite B cell activation factors, induces activation-induced cytidine deaminase (AID) expression, which is required for both CSR and SHM [57]. Furthermore, induction of AID is driven through the transcription factor STAT6 [58]. Recently, a novel cytokine reporter system in mice allowed visualization of the temporal spatial distribution of IL-4 during GC development. It was observed that IL-4 secretion by activated T cells was restricted to the follicles, and later the GC, and that T cells with increased IL-4 secretion were positive for the T_FH_ markers, CXCR5 and ICOS (inducible T-cell co-stimulator) [59]. Again, there was an increase in AID transcript levels in T-B cell conjugates when the component T cells were expressing high levels of IL-4. However, the study showed that IL-4 was not required for GC formation [59]. The main role of IL-4 appears to lie in the regulation of CSR and SHM.

IL-6 is another cytokine that has been implicated in having a role in GC reactions and subsequent PC generation. Early on, IL-6 was shown to induce Ig production in human B cells [60]. However, more recently it appears that IL-6’s role may lie within signaling T_FH_ cells, as well as (and perhaps more so than) B cells in the GC reaction [61]. FDCs in the GC are positive for IL-6 secretion and one study, using IL-6 knockout mice, was able to show that IL-6 at least enhanced GC formation and plays a role in SHM [62]. A subsequent study demonstrated a requirement for IL-6 in the eradication of lymphocytic choriomeningitis virus (LCMV) due to a late spike in IL-6 production, 25 days post infection [61]. The same study demonstrated that signaling through T_FH_ was responsible for that effect by using IL-6 conditional knockout mice [61].

IL-10 also plays a role in signaling that leads to B cell differentiation in the GC. It appears that IL-10 is a key driver of PC differentiation *vs.* memory B cell formation in the final stages of the GC reactions [63]. Treatment with IL-10 leads to differentiation to PCs and its absence results in GC B cells that favor the memory B cell lineage [64].

Just as BLIMP-1 is the master transcriptional regulator of PC generation, IL-21 seems to have emerged as perhaps the master cytokine of GC formation and subsequent PC differentiation. As was stated above, IL-2 has been shown multiple times to induce PC generation [54,55,56]. However, in human B cells, treatment with IL-21 alone in combination with CD40L and anti-IgM enhances PC generation to a higher degree than cells treated with IL-2 alone [56]. Furthermore, IL-21 positively regulates BLIMP-1 and results in increased Ig secretion of IgG and IgM [56]. A study in mice also indicates that IL-21 deficiency results in diminished GC formation and a decrease in the numbers of both T_FH_ cells and GC B cells, with a greater reduction seen in the B cell population [65]. Mechanistically, signaling favoring PC differentiation from IL-21 seems to go through STAT3-mediated upregulation of BLIMP-1 and IRF4 [21,22,66]. One recent study, showed that STAT3 directly binds the BLIMP-1 promoter and competitively inhibits the binding of BCL6 [22]. Since BCL6 has a mutual repressive relationship with BLIMP-1, this should result in increased expression of BLIMP-1 and decreased expression of BCL6. Furthermore, the study shows that STAT3 binds to the BLIMP-1 3' intron, which is believed to be the mechanism of BCL6 mediated inhibition of BLIMP-1 [22]. In support of this evidence, an interesting phenotype develops when BCL6 overexpressing cells are treated with IL-21. These cells upregulate BLIMP-1, while maintaining BCL6 levels, which results in differentiation to high Ig secreting, but also highly proliferative cells [21]. This phenotype is seen in lymphocytic malignancies that are discussed in greater detail below.

Chemokines and their receptors also play a role in GC formation and subsequent PC development. As stated above, prior to GC formation, Ag activated B cells migrate to the T-zone of the lymph node where they complex with CD4^+^ T cells. Chemokine C-C motif receptor 7 (CCR7) and its ligand, chemokine C-C motif ligand 21 (CCL21), are necessary for this migration. B cells from CCR7 knockout mice did not migrate to the T zone. Furthermore, a CCL21 gradient existed, with the highest concentrations found at the T zone [67]. During the GC reaction, chemokine C-X-C motif receptor 4 (CXCR4) and chemokine C-X-C motif receptor 5 (CXCR5) are required for appropriate organization of the dark and light zones. Mice deficient in CXCR4, as well as treatment of cells with a CXCR4 inhibitor, resulted in unorganized GCs [68]. Furthermore, CXCR4 expression on centroblasts was increased and was required for migration to the dark zone of the GC. Mice deficient in CXCR5 or its ligand, chemokine C-X-C motif ligand 13 (CXCL13), still formed light and dark zones but with atypical organization [68]. A subsequent study demonstrated, in human cells, that CXCR4^+^ cells had increased mRNA expression of BCL6 and BACH2, whereas CXCR4^−^ cells had increased BLIMP-1 and IRF4 mRNA expression, as well as increased IgG secretion [69].

All in all, it is abundantly clear that signaling between the cells comprising the GC is paramount to appropriate affinity maturation, proliferation, and differentiation. However, the intricacies of this signaling system both temporally and spatially are yet to be deduced.

## 6. Role of the BM Niche in the Production of Ig

Long-lived PC primarily reside in the BM, where they can persist for nearly a lifetime and continually secrete high affinity Ig, as well as help provide immunological memory [3,70]. This remarkable longevity is facilitated by signals from the BM microenvironment. Upon leaving the GCs in either the lymph nodes or the spleen, PCs upregulate CXCR4, whose ligand CXCL12, is produced in the BM and is used as a homing mechanism for these PCs [71]. Homeostasis of BM PCs is maintained through complex interactions, in what have been termed bone marrow niches, between the PCs and resident cells of the BM.

PC homeostasis and subsequent Ig secretion in the BM is facilitated in part by paracrine cytokine signaling. One of the key cytokines involved in PC survival and subsequent Ig secretion is IL-6 [72,73,74,75,76,77,78,79,80]. PCs isolated from the BM of mice showed that PC numbers depleted when grown in media alone as opposed to the BM suspension [79]. Further, increased survival of BM PCs was not maintained in BM suspension obtained from IL-6 knockout mice [79]. A similar mechanism is observed in human BM PCs. IL-6 blockade leads to decreased Ig secreting PCs and treatment with exogenous IL-6 has a rescuing effect [78]. These studies demonstrate a strong role for IL-6 in the maintenance of Ig secreting PC numbers in the BM.

Another set of key cytokines involved in BM PC maintenance, are those of the tumor necrosis family, APRIL (a proliferation inducing ligand) and BAFF (B cell activating factor) [81,82]. BAFF can bind to three different receptors, BAFF receptor (BAFFR), which it activates exclusively, and transmembrane activator and CAML (calcium-modulator and cyclophilin ligand) interactor (TACI) and B cell maturation Ag (BCMA), which also share APRIL as a ligand [82,83]. Multiple studies have shown that BCMA and TACI are expressed on PCs in mice and humans [71,76,84,85]. In mice, an increase in BCMA and TACI is seen following immunization [71]. Furthermore, blockade of TACI and knockout of BCMA leads to decreased Ab secreting cells [76]. Most studies implicate APRIL as the major player in promoting PC survival between the two cytokines, although BAFF has also been shown to enhance survival and Ig secretion to some degree [76,80,84,86,87]. APRIL knockout mice exhibit decreased PCs and decreased serum Ig titers [87]. Using human PCs, a combination of APRIL and IL-6 further enhanced PC survival and Ig levels [80]. Together, these studies indicate that APRIL is a key component of maintaining the long-lived PCs in the bone marrow.

There is a good deal of evidence suggesting that the source of these PC promoting cytokines come from a number of different cell types found in the bone marrow. Although the full extent of the cross-talk between BM PCs and other cells of the microenvironment is not fully understood, a great deal of progress has been made in the last decade in unraveling this complex system of paracrine signaling.

One of the foremost cells involved in the homeostasis of PC’s are BM stromal cells. It is unclear whether direct contact between BM stromal cells and PCs is required in BM PC maintenance. In one study, blockade of adhesion molecules in mice leads to a decrease in IgG1 secretion and it was also shown that stromal cells produce IL-6 which, as discussed above, is required for PC homeostasis [73]. However, more recently it was shown in mice that the effect of stromal cells on PC survival is not contact dependent using an *in vitro* system where PC’s were cultured in transwells with stromal cells [88]. The pro-survival effects of stromal cells are equivalent to the effects of treating PCs with a combination of exogenous IL-6 and APRIL [80].

Other than stromal cells, the bone marrow is composed of numerous types of immune cells and is the site of hematopoiesis [89]. A number of these immune cells have been implicated in providing the necessary signals for PC survival. One study demonstrated the ability of basophils to positively affect PC Ig secretion [74]. Basophils enhanced IgG production 17 days post immunization and increased IL-6 secretion from the basophils is thought to be a probable cause of the increased IgG [74]. Furthermore, the depletion of basophils *in vivo* resulted in decreased total serum Ig [74].

Activated eosinophils secrete several cytokines including IL-6, IL-10, and APRIL and in mice, a steady increase in eosinophils in the bone marrow occurs up to 100 days after immunization [75]. As would be expected, co-culture of PCs with activated eosinophils results in increased survival and conversely, depletion of eosinophils in the bone marrow results in decreased PC survival [75,90]. Interestingly, megakaryocytes present in the BM have been shown to produce large amounts of IL-6 [77]. However, depletion of megakaryocytes in mice led to a decrease in IgA secretion while it had no effect on IgG or IgM secretion *in vivo* [77].

The myeloid lineage cells, osteoclasts (OC) are responsible for bone resorption and are involved in hematopoiesis [91]. *In vitro*, osteoclasts are able to maintain PC survival better than dendritic cells (DCs). Furthermore, cell-cell contact is required and neutralizing BAFF and APRIL had no effect on OC mediated PC survival [92]. An interesting study was able to link the prolonged survival of PCs with hematopoietic development of granulocytes [86]. Using a system able to detect APRIL secreting cells, the study determined that the major producers of APRIL in human cells are myeloid precursors [86]. The myelocytes are rarely in proximity to the PCs. Rather, they secrete APRIL that flows freely through the bone marrow and is readily taken up by PCs [86]. Interestingly, contrary to what has been shown in mice, megakaryocytes proved to be negative for APRIL secretion in human BM aspirates [86].

Not only do paracrine signals from a network of cytokine secreting cells mediate PC survival and Ig secretion, but also there are direct cell-cell interactions that are known to play a role in this process. One such interaction is the interaction between CD28 and its ligands CD80 or CD86. These markers are generally associated with T cell activation [93]. However, CD28 has also been found to be expressed on PCs [72]. CD28 deficient mice had reduced serum Ig titers and the reduction appears to be due to PC intrinsic signaling through CD28 and not a result of deficiency in activating T cells [72]. Furthermore, the Ig inducing signaling comes from an interaction with BM dendritic cells (DCs) in which ligation of PC CD28 and DC CD80/86 results in increased production of IL-6 by DCs, which in turn promotes PC survival and Ig secretion [72]. Accordingly, when BM PCs were cultured with BM DCs deficient in IL-6, Ig production was nearly completely abolished [72]. Interestingly, the same study was able to show that activation of the NF-κB pathway through CD28 signaling occurs only in BM PCs and not in short-lived splenic PCs [72]. The surface marker CD93 is expressed on developing PCs as well as long-lived PCs of the bone marrow. CD93 is also expressed on myeloid and endothelial cells and the soluble form has been shown to potentiate angiogenesis in mice [94,95]. Mice deficient in CD93 exhibit impaired Ab production following immunization [96]. Overall, long-lived PC survival in the BM requires multiple external signals, both from paracrine sources and cell-cell contact.

The result of the above signaling events is continued activation of proteins involved in PC differentiation, as well as upregulation of anti-apoptotic proteins. One study showed that culture of human PCs with either stromal cells or a combination of IL-6 and APRIL leads to increased transcripts of IRF4, BLIMP-1, and XBP-1 [80]. Interestingly, a slight increase in BCL6 also occurs between day 30 and day 60 in PCs grown *in vitro* [80]. Of note, in another study, that also developed long lived PCs *in vitro*, the spliced variant of XBP-1 decreases once the ER has expanded and the secretory burden has been relieved [88].

In addition to the regulation of factors involved in PC differentiation, the anti-apoptotic factor, myeloid cell leukemia 1 (MCL-1) is activated in mice through BCMA signaling [76,85]. Furthermore, mice with conditional MCL-1 knockout have much lower Ab titers post immunization than WT mice [85]. The anti-apoptotic proteins, BCL-2 and BCL-xL have also been shown to be upregulated in murine BM PCs [85,87]. Moreover, treatment with APRIL resulted in increased BCL–xL levels [87]. The role of these different anti-apoptotic factors, as well as other upstream activators in healthy human PCs has not yet been determined.

The intricacies of PC survival in the BM are still being uncovered. However, the above events all appear to play some role in the ability of PCs to persist in the BM and continually secrete Ab throughout the lifetime of the host. This is tremendously important in an organism’s ability to develop an immunological memory that confers a more efficient response to secondary infections.

## 7. Regulation of Ig in Lymphoid Malignancies and Autoimmune Disease

The complexity of the mechanisms regulating Ig secretion in PCs is most likely a product of the need for tight regulation governing the development of PCs. Dysregulation of these events can lead to autoimmune disease as well as malignant disease (Table 1).

Sjӧgren’s Syndrome (SS) is a systemic autoimmune disease characterized by hypergammaglobulinea and circulating autoantibodies. Symptoms associated with SS are decreased tear production and lowered rates of salivary flow [9]. Multiple studies have shown the development of ectopic GCs within salivary glands of SS patients [97,98,99]. In one of the studies, PCs were found near the GC as well as clustered near acinar and ductal epithelial cells [97]. Increased CXCL12 expression on ductal epithelial cells likely plays a role in the clustering of PCs to these areas [100]. Patients positive for GCs showed increased levels of serum IgG [97]. In a subsequent study, B cells from salivary gland GCs had increased transcript levels of AID and BCL6, indicating that these ectopic GCs could possibly be functional. Furthermore, autoreactive B cells were not excluded from salivary gland GCs [98].

As would be expected, cytokines also appear to play a role in the pathogenesis of SS. Mice constitutively overexpressing BAFF develop enlarged and inflamed salivary glands. Likewise, serum BAFF levels are elevated in patients with SS [101]. In humans, increased levels of BAFF correlate with elevated total IgG and IgM, as well as elevated autoreactive Abs [102]. More recently, overexpression of BAFF in healthy mice led to increased autoantibody production [103]. Clearly, dysregulation of processes normally involved in PC differentiation and survival plays a role in the pathogenesis of SS.

Multiple myeloma (MM) is a PC malignancy that is nearly always preceded by monoclonal gammopathy of undetermined significance (MGUS) [11]. It is characterized by increased numbers of malignant PCs in the BM and high serum levels of monoclonal Ab [11]. The origin of the MM cells appears to be from GC PCs as MM cells have mutated variable regions and also have undergone CSR [104]. In MM, clonotypic malignant cells most commonly express IgG or IgA, but pre-switched IgM cells are also found [105]. Elevated levels of Ig in MM patients can lead to renal failure, as well as immunodeficiency due to outcompetition of MM with normal PCs, thus leading to decreased levels of immunologically active Ig in the serum [11]. Currently, there is no cure for MM, although novel therapies are quickly emerging [106].

**Table 1:** Proteins involved in dysregulated Ig production in autoimmune and malignant diseases. Proteins involved in dysregulated Ig production in the autoimmune disease, Sjӧgren’s syndrome, and the malignant diseases, multiple myeloma and Waldenstrӧm macroglobulinemia.

Disease	Name	Type	Role	Refs
**Sjӧgren’s Syndrome**	BCL6	Transcription factor	Formation of ectopic GCs and increased serum IgG	[96,97]
	BAFF	Cytokine	Increased Ig Secretion	[100,101,102]
	CXCL12	Chemokine	Clustering of PCs to acinar and ductal epithelial cells	[99]
**Multiple Myeloma**	MYC	Transcription factor	Activation mediated by IRF4 or due to *IGH* translocation leads to proliferative phenotype	[107,108]
	IRF4	Transcription factor	Activation of MYC	[107]
	XBP-1s	Transcription factor	Elevated levels lead to decreased progression-free survival and increased serum Ig in mice	[109]
	IL-6	Cytokine	Survival and proliferation	[106]
	BAFF	Cytokine	Survival and proliferation	[106]
	APRIL	Cytokine	Increased proliferation by activation of cyclin D2	[108]
**Waldenstrӧm Macroglobulinemia**	GLI2	Transcription factor	Regulation of IL-6Rα in WM cells and regulation of IL-6 in stromal cells leading to modulation of IgM secretion	[110,111]
	BAFF	Cytokine	Increased IgM secretion	[112]
	IL-21	Cytokine	Increased IgM secretion and increased expression of BLIMP-1 and XBP-1	[113,114]
	IL-6	Cytokine	Increased IgM secretion	[110,115]
	CCL5	Chemokine	Leads to GLI2 activation in stromal cells	[110,115]

As was the case with normal long-lived PCs, signaling in the BM microenvironment plays a significant role in the pathogenesis and progressiveness of MM. MM cells have elevated levels of BCMA, BAFF-R, and TACI and it is been shown that treating MM cells *in vitro* with IL-6 and BAFF leads to an increase in survival, which is similar to what is found in normal PCs [116]. Contrary to events in normal PCs, treatment of MM cells with these cytokines also leads to an increase in cell proliferation [116]. Using an *in vivo* MM mouse model, increasing levels of soluble BCMA leads to a significant increase in serum Ig titers [117]. Likewise, blockade of BCMA results in a decrease in the viability of MM cells *in vivo* [118].

As with many cancers, chromosomal translocations are a significant source of genomic aberrations in MM [107,119]. Many of the translocations found in MM patients occur within the *IGH* gene in which the transcriptional regulation of oncogenes, such as v-myc avian myelocytomatosis viral oncogene homolog (MYC), are located under the control of Ig enhancers [108]. This leads to the hijacking of PC homeostatic signals that would normally result in continued Ig secretion but instead, lead to proliferative phenotypes [108,120]. One study found that treatment of MM cells with APRIL led to upregulation of cyclin D2 and an increase in proliferation [120]. The effect of APRIL was differential in MM cells depending on their translocation status [120]. Another study demonstrated a positive feedback loop where IRF4, a normal regulator of PC differentiation, upregulated MYC, which in turn continued to upregulate IRF4 [108]. In addition to IRF4, other key regulators of long-lived PC development have been implicated in MM. BLIMP-1 mRNA was elevated in MM PCs. Furthermore, MM B cell population had high levels of BLIMP-1 transcript compared to low to undetectable levels in normal B cells [109]. Elevated levels of spliced XBP-1 (XBP-1s) in MM patient samples led to the development of a MM mouse model with B lineage cells constitutively overexpressing XBP-1s. Using this model, high levels of XBP-1s led to decreased progression free survival and increased serum Ig levels [121].

Waldenström macroglobulinemia (WM) is a lymphoplasmacytic lymphoma, which, like MM, is most often preceded by MGUS in patients [122]. One of the major complications with WM is increased serum levels of IgM, which can lead to serum hyperviscosity in patients. [122]. Unlike MM, WM cells have not undergone CSR and only secrete IgM [112,123]. They do exhibit SHM, suggesting that Ag exposure and the GC reaction may be the source malignant B cells [112,123]. WM cells can be found circulating in the blood and lymphoid tissues but primarily home to the BM [112]. Given the clinical complications caused by elevated levels of IgM, it is of great importance to determine the regulation of IgM production in WM.

Similar to MM and normal PCs, the crosstalk between WM cells and resident cells of bone marrow play a large role in disease progression and enhancement of IgM secretion. As with MM, WM cells express BAFFR, BCMA, and TACI and patients with WM have elevated levels of BAFF [113]. WM cells cultured *in vitro* show increased IgM secretion when treated with BAFF and a combination of cytokines that are important for regulation of Ig secretion in normal B cells, compared to cells treated with cytokines alone, demonstrating a potential role for BAFF in the regulation of IgM secretion [113]. More recently, IL-21 was shown to increase IgM secretion in WM cells through a STAT-dependent mechanism [120]. Treatment with IL-21 also led to increased expression of BLIMP-1 and XBP-1 [115]. Another study showed that IL-6 led to increased secretion of IgM but had no effect on survival in WM cells [110]. Using a coculture system it was shown that treatment with CCL5 resulted in increased IL-6 secretion by WM stromal cells which in turn resulted in increased IgM secretion by WM cells [110]. Interestingly, CCL5 induction of IL-6 secretion was shown to be mediated through non-canonical activation of the oncogene, glioma associated protein 2 (GLI2) [124]. Therefore, these studies highlight the important role of signaling events in the tumor microenvironment in promoting Ig secretion by malignant cells. More recently, our group has identified GLI2 as a potential regulator of IgM secretion in WM cells. Regulation of IgM secretion appears to most likely be occurring via GLI2’s activation of IL-6Rα [111].

## 8. Conclusions

Clearly, our understanding of factors regulating Ig production by PCs has greatly increased over the last couple of decades. However, our understanding of regulatory factors involved in its production in malignant disease remains poorly understood. Given the clinical complications that arise from the heightened Ig production in malignant B cells/plasma cells, it is of great importance that we continue to expand our knowledge of the processes regulating Ig expression and secretion in order to develop new therapeutic approaches that can target these processes.
